# DNA circuits driven by conformational changes in DNAzyme recognition arms†

**DOI:** 10.1039/d0ra00115e

**Published:** 2020-02-24

**Authors:** Xinyi Sun, Xuedong Zheng, Sue Zhao, Yuan Liu, Bin Wang

**Affiliations:** Key Laboratory of Advanced Design and Intelligent Computing, Ministry of Education, School of Software Engineering, Dalian University Dalian 116622 China wangbinpaper@gmail.com; College of Computer Science, Shenyang Aerospace University Shenyang 110136 China xuedongzheng@163.com; School of Computer Scicence and Technology, Dalian University of Technology Dalian 116024 China Liuyuan.dlut@gmail.com

## Abstract

DNA computing plays an important role in nanotechnology due to the unique programmability and parallelism of DNA molecules. As an important tool to realize DNA computation, various logic computing devices have great application potential. The application of DNAzyme makes the achievements in the field of logical computing more diverse. In order to improve the efficiency of the logical units run by DNAzyme, we proposed a strategy to regulate the DNA circuit by the conformational change of the E6-type DNAzyme recognition arms driven by Mg^2+^. This strategy changes the single mode of DNAzyme signal transmission, extends the functions of E6-type DNAzyme, and saves the time of signal transmission in the molecular scale. To verify the feasibility of this strategy, first, we constructed DNA logic gates (YES, OR, and AND). Second, we cascade different logic gates (YES–YES, YES–AND) to prove the scalability. Finally, a self-catalytic DNA circuit is established. Through the experimental results, we verified that this DNAzyme regulation strategy relatively reduces the cost of logic circuits to some extent and significantly increases the reaction rate, and can also be used to indicate the range of Mg^2+^ concentrations. This research strategy provides new thinking for logical computing and explores new directions for detection and biosensors.

## Abstract 2

DNAzyme recognition arms conformational changes drive DNA nanoscale logic circuits without the need for cleavage.

## Graphical abstract

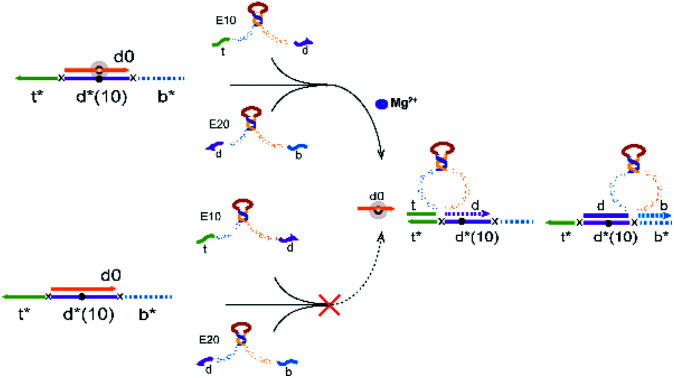

## 1 Introduction

DNA molecules, which follow the principle of complementary base pairing, have the characteristics of programmability and high-density storage, and have gradually become a hot research material. DNA materials are used in combination with DNA nanotechnology, such as strand replacement,^1^ DNA tiles,^2^ origami,^3^ nanoparticle assembly with DNA,^4^*etc.*, has made great achievements in the fields of biochemistry, computing, information and medicine.^5^ Based on these technologies, people have designed DNA switches,^6^ DNA regenerators,^7^ encryption systems,^8^ detection devices,^9^ nano-robots^10^ and other DNA driven devices. This is specific application of a nano-logic circuit,^11^ one of the research hotspots due to its great potential in logic calculation,^12^ data processing^13^ and information sensing,^14^ biochemical reaction network,^15^ neural networks^16^ and catalytic reaction networks^17^ and other modules practically.

In order to enrich the functions and varieties of logic circuits, people are exploring more and more ways to realize DNA logic circuits. Due to its high specificity and catalytic efficiency, enzymes, including restriction endonuclease^18^ and DNAzyme,^19^ are often used as DNA strand cutting tools to participate in logic DNA computing,^20^ providing technical support in DNA computing, which is commonly used in DNA computing.^21^ Specifically, DNAzyme is widely used in the construction of DNA logic circuit because of its simplicity and similarity with DNA strands in reaction conditions.^22^ In the basic units of the logic circuits, DNAzyme can build switch modules triggered by metal ions^23^ DNAzyme is combined with ordinary DNA strands to establish a universal logic gate module, which provides a reference for the assembly of Boolean logic units.^24^ At the same time, DNAzyme can also be combined with caged G-quadruplex to build molecular logic gates for label-free diagnostics,^25^ which have laid the foundation for the expansion of logic circuits. Further, DNAzyme participates in the construction of large-scale logic circuits, including logic operation circuits,^26^ combinational cascade circuits,^27^ feedback circuits,^28^ and catalytic cycle circuits^29^ to achieve information processing of different complexity.

As a special DNA molecule with a specific sequence, DNAzyme has a variable and controllable structure and can cleave specific substrates. DNAzyme played an important role in the construction of logic circuits and contributed new ideas to the development of logic computing. DNAzyme is a metal-dependent enzyme that can undergo conformational changes under the control of ions. That is, in the presence of ions, DNAzyme will fold globally, forming a specific stem-loop structure. If the metal ion concentration is enough, DNAzyme will further promote the hydrolysis of ribose phosphate bond to achieve cleavage,^30^ expose toehold, or produce another DNA strand, which then drives the downstream reaction. In common logic circuits constructed by DNAzyme and DNA strands,^24^ the role of DNAzyme is to cut specific DNA single strands in order to complete signal generation and transmission.^25^ RNA must be modified on specific single-stranded substrates to ensure successful cleavage. In order to simplify the reaction conditions of DNAzyme in the circuit and expand its function, we have adopted a more direct approach to achieve signal transmission, in which Mg^2+^ was used to regulate^31^ the conformation of DNAzyme to generate output signal through a strand displacement reaction.^32^ Firstly, we omit the cleavage process, mainly considering the possibility of binding the recognition arm to the substrate and do not need to consider the effect of the C/G content in the recognition arms and the hairpin structure on the cutting rate, which reduces the difficulty and complexity of the experiment and relatively saves the time consumed to produce the output. Secondly, the specific recognition substrates of DNAzyme are no longer limited to single strands. DNAzymes can react with the double-stranded DNA molecules that have at least one toehold, which signals can be transmitted, and thus provide more ways for the cascades of biochemical circuits constructed by strand displacement technology. Thirdly, the circuits can also indicate the range of ion concentrations. We replaced the RNA base with the DNA bases. Compared with the method of detecting ion concentration through DNAzyme cutting, the material cost is relatively reduced and the reaction speed may be improved, laying the foundation for a new model of ion sensing. We try to explore more ways to use DNAzyme and enrich the achievements in the field of DNA nanotechnology.

In that work, we chose to study the characteristics of E6-type DNAzyme, designed and implemented a new way of DNA logic circuit system that utilizes the conformational changes of E6-type DNAzyme recognition arms. We build basic logic gates (YES gate, OR gate, and AND gate) to prove the possibility of this strategy to build multiple logic gates. In order to demonstrate the scalability and applicability, we constructed YES–YES cascade, YES–AND cascade and self-catalytic DNA circuit. The conformation of E6-type DNAzyme changes under the drive of Mg^2+^. In that process, discontinuous recognition arms act like continuous DNA strands, which can hybridize to double-stranded substrates *via* a toehold domain, to generate new DNA strands to trigger downstream reactions. In this way, the reaction, that the E6-type DNAzyme recognition arms participate in, can achieve a similar effect to cleavage, while saving the time for cleavage, which hydrolysis reaction requires. What's more, we diversify the strand reaction of DNA circuit, which will happen when DNAzyme cleavage the substrate for triggering the downstream reaction, as well as the way for its signal delivery, and its function in circuit, which will not only reduce the difficulty and complexity of operation, but also improve the efficiency of E6-type DNAzyme in biochemical reactions. In terms of materials, RNAs that modify specific substrates can be replaced by DNA bases, reducing the cost of logical operations to some extent. Native polyacrylamide gel electrophoresis and a fluorescence assay were used to verify the feasibility and robustness of E6 DNAzyme conformational changes to regulate DNA circuit strategies.

## 2 Results and discussion

### 2.1. Basic YES gate controlled by DNAzyme conformational changes

**Fig. 1:**
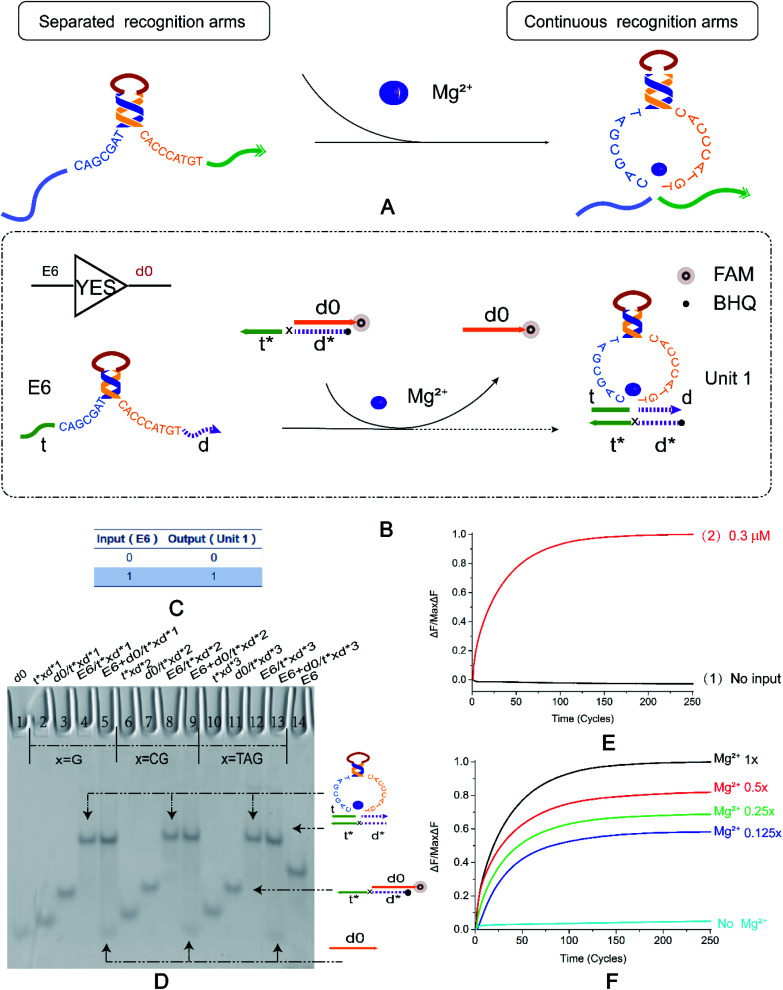
(A) The DNAzyme recognition arms undergo a conformational change under the control of Mg^2+^. (B) YES logic gate reaction schematic. The 3′ end of d0 in the double-stranded substrate modifies the fluorophore FAM, and the 5′ end of t*d* modifies the quenching group BHQ. The E6 DNAzyme recognition arms undergo a strand displacement reaction with a double-stranded substrate and binds to t*d* therein to release a fluorescent signal. (C) YES logic gate corresponding to the logical operation truth table. (D) Native PAGE analysis of the YES logic gate. The number is marked above each lane, and the top is the DNA strand type for each lane. Lane 1: d0; lane 14: E6 DNAzyme; lane 1: d0; lane 14: E6 DNAzyme; The 2–14 lanes are divided into three parts, which are three implementation methods of the YES logic gate. The bases of the “x” in the DNA strand t*xd * are “G”, “CG”, and “TAG”. Lanes 2, 6, and 10 are t*xd*; lanes 3, 7, and 11 are the double strands synthesized by t*xd* and d0 ([t*xd*] : [d0] = 1 : 1.1). Lanes 4, 8 and 12 are structures formed by annealing E6 with t*xd* ([E6] : [t*xd*] = 1 : 1.1), and are also strands for comparison with the results. Lanes 5, 9, and 13 are the logical gates for the reaction of the E6 DNAzyme with the double-stranded substrate ([E6] : [t*xd*/d0] = 1 : 1). (E) YES logic gate normalized to the fluorescence intensity curve, solution concentration is 0.3 μm, sampling scan interval time was set to 6 s, for a total of 251 cycles. (F) Normalized fluorescence curve of YES logic gate fluorescence intensity as a function of Mg^2+^ concentration. All data represent the average of three replicates, and the error bar represents one standard deviation representing three replicated analyses.

In order to extend the functions of E6-type DNAzyme and optimize its efficiency in biochemical reactions, we seek breakthrough points based on its conformational changes. Fig. 1A shows that the E6-type DNAzyme is a DNA single strand with a part of a specific base sequence (*i.e.*, a conserved domain) in solution. E6-type DNAzyme undergoes a conformational change in the presence of metal Mg^2+^. The separated recognition arms at both ends converge to bind to a specific substrate. If no Mg^2+^ is added to the buffer, then the E6-type DNAzyme will still be in a single-stranded state and free in solution. The E6-type DNAzyme bound to the substrate releases a new DNA strand through a hybridization reaction. In this process, the E6-type DNAzyme does not need to undergo a hydrolysis reaction to generate the substrate, and completes signal transmission. To prove the feasibility of this operation, we designed the basic YES logic gate.

The YES logic gate (Fig. 1B) is triggered by Mg^2+^. The input part is E6-type strand, and the recognition arms at both sides are t and d. The other part is a double-stranded substrate called d0/t*xd*, and the 3′ end of d0 modifies the fluorophore. The strand t*xd* is the binding strand of the DNAzyme recognition arms, and its 5′ end modifies the quenching group, and the 3′ leaves a toehold. The “x” site shown in the figure represents the DNA bases in the strand t*xd*. The ribonucleobase “rA” in the cleavage site “TrAGG” is replaced by the “x” site, changing from “TrAGG” to “TxGG”. Specific substrates are no longer modified by RNA. In the presence of Mg^2+^, E6-type DNAzyme undergoes conformational changes. The strands t and d become continuous, which then binds to toehold t* and then binds to d*, releasing d0 that has been modified with a fluorophore. In the absence of Mg^2+^, the input E6-type DNAzyme and the double strand are independent of each other and do not react.

First, we used basic polyacrylamide gel electrophoresis to verify the experimental results. As shown in Fig. 1D, we replaced the part that was originally modified by RNA with three forms of DNA bases (Fig. 1D, X = G, X = CG, X = TAG). After adding E6-type, it reacts with the double strand d0/t*xd* (Fig. 1D, lane 3, lane 7, lane 11), and the result is two bands (Fig. 1D, lane 5, lane 9, lane 13); one lower band is at the same horizontal line as lane 1, and one upper band is on the same horizontal line as the complex formed by annealing of E6-type enzyme and t*xd* (Fig. 1D, lane 4, lane 8, lane 12). It was proved that the E6-type DNAzyme completed the reaction as expected and output the correct signal.

To better demonstrate the process of signal output, real-time fluorescence detection was also performed (Fig. 1E and F). As shown in Fig. 1E, when the input strand E6-type is added, a fluorescent signal that gradually increases in intensity (Fig. 1E, curve 2) and subsequently stabilizes was observed, and a significant change in fluorescence intensity was not detected in the absence of E6-type (Fig. 1E, curve 1). The fluorescence results also demonstrate the successful implementation of the YES logic gate. Fig. 1F shows that Mg^2+^ concentration in the buffer was adjusted to 12.5 mM of the standard data and 1/2, 1/4, 1/8 of 12.5 mM (corresponding to 1×, 0.5×, 0.25× and 0.125× in Fig. 1F respectively), in the case where the concentration of the reaction strand in the solution was constant. In the absence of Mg^2+^, the fluorescence intensity does not significantly increase and as the Mg^2+^ concentration increases, the fluorescence intensity curve is also gradually enhanced. It is indicated that the concentration of Mg^2+^ ions has different degrees of adjustment to the allosteric structure of DNAzyme, which affects the signal output of the YES logic gate and can roughly reflect the concentration range of the Mg^2+^. Native PAGE without Mg^2+^ and the effect of input strand concentration on results is shown in the ESI (Fig. S2†).

### 2.2. Basic AND gate controlled by DNAzyme conformational changes

**Fig. 2:**
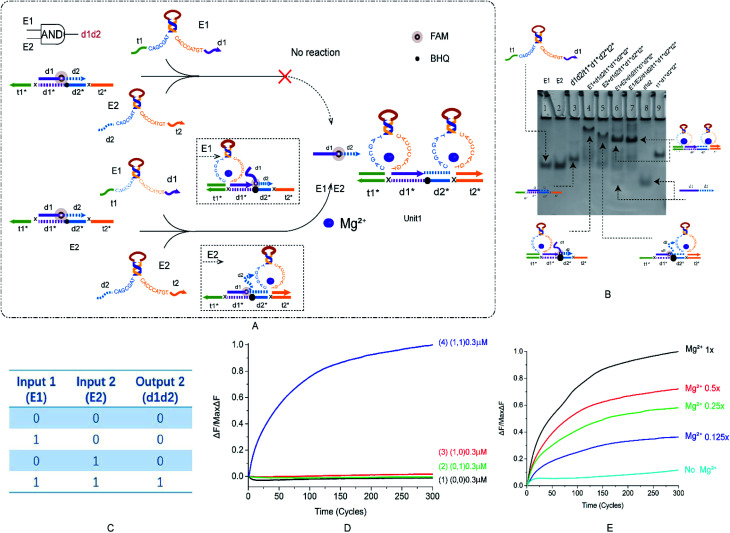
(A) The schematic diagram of the AND logic gate. The fluorophore FAM modification is in the middle of d1d2, the annihilation group BHQ is modified in the middle of the t1*d1*d2*t2* strand. E1 and E2 react from both sides of the double-stranded substrate and replace the fluorescent strand. (B) Native PAGE analysis of the AND logic gate. The number is marked above each lane and the top is the DNA strand type for each lane. Lane 1: DNAzyme E1; lane 2: DNAzyme E2; lane 3: double strand formed by annealing of strand d1d2 with strand t1*d1*d2*t2* ([d1d2*] : [t1*d1*d2*t2*] = 1.2 : 1); lane 4: reaction with AND logic gate when input is DNAzyme E1; lane 5: reaction with AND logic gate when input is DNAzyme E2; lane 6: reaction with AND logic gate when input is DNAzyme E1 and E2 ([E1] : [E2] : [t1*d1*d2*t2*] = 1 : 1 : 1); lane 7: structure formed by annealing of DNAzyme E1, E2 and t1*d1*d2*t2* ([E1] : [E2] : [t1*d1*d2*t2*] = 1 : 1 : 1); lane 8: strand d1d2; and lane 9: strand t1*d1*d2*t2*. (C) AND logic gate corresponding to the logical operation truth table. (D) Normalized fluorescence intensity curve of the AND logic gate; the solution concentration is 0.3 μm, and the sampling scan interval is set to 10 s for a total of 300 cycles. (E) Normalized fluorescence curve of the AND gate logic fluorescence intensity as a function of Mg^2+^ concentration.

We want to verify the versatility of this principle and prepare for the construction of multi-level cascade circuits. After building the most basic YES logic gate, we started to construct the OR and AND logic gates. The specific reaction of AND is shown in the Fig. 2A, which is divided into two modules: Mg^2+^ trigger and no Mg^2+^ trigger. As shown in Fig. 2A, E6-type DNAzymes E1 and E2 were used as input and reacted with a double strand with a toehold at both sides. Their recognition arms combine to bind the long-strand bases in the double-stranded substrate. The reaction process is regulated by switching with Mg^2+^. The fluorophore and the quenching group are modified in the middle of the double strand. The truth table of the AND logic gate is shown in the Fig. 2B. If and only if both enzymes E1 and E2 are present, then the output of the result signal will be available. In other cases, the output is 0.

During the design of the double-stranded substrate that combines with two DNAzymes, two problems were addressed. One is that if the AND gate is designed using the recognition arms of the YES and OR logic gates, then the ratio of DNAzyme to double-strand binding will be small. This problem is resolved by increasing the content of the recognition arms bases C and G. Another was found that when the short-strand d1d2 was completely combined with the long-strand t1*d1*d2*t2*, the output signal d1d2 generated was particularly small, and an ideal output was not obtained. The difficulty of releasing d1d2 was solved by adding a base-length bubble in the middle of d1d2. More detailed instructions are included in the ESI (Fig. S4†). After modification, the AND logic gate is implemented.

The results of PAGE are shown in Fig. 2B. Lanes 3–6 are (0, 0), (1, 0), (0, 1), and (1, 1), respectively. Lanes 7 and 8 are two sets of comparative DNA strands. The figure shows that only (1, 1) can get the ideal output signal (Fig. 2C, lane 6). The results of fluorescence analysis show that as the two inputs increase, the fluorescence intensity gradually rises (Fig. 2D, curve 4), and in other cases, the fluctuation is small (Fig. 2D, curve 1–3), which also conforms to the expected conclusion. Gradient comparison of Mg^2+^ concentration shows that the AND gate fluorescence curve has a larger distribution pitch than the YES logic gate and is more sensitive to changes in Mg^2+^ concentration, which can better reflect the Mg^2+^ concentration range (Fig. 2E). Native PAGE without Mg^2+^ and the effect of input strand concentration on results is shown in the ESI (Fig. S3†). The OR gate is presented as ESI† and will not be described here (Fig. S3†).

In this study, the characteristic of the basic logic gates is that E6-type DNAzyme does not choose the conventional way of cutting specific substrates to generate output signals. It uses a structural change in the recognition arms and then hybridizes with a specific substrate to release a signal. Compared to the allosteric DNAzyme-based DNA logic circuit described by Xuedong Zheng and Jing Yang,^28^ this change in signal transmission mode shortens the action time for E6-type DNAzyme and is easier to control. Next, we verify that this strategy works as expected in a logic cascade circuit.

### 2.3. Two-stage YES–YES cascade circuit

**Fig. 3:**
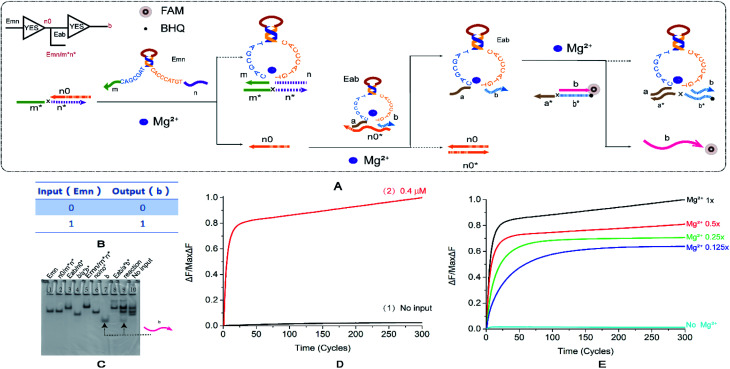
(A) Reaction schematic diagram of the YES–YES cascade logic circuit. The fluorophore FAM is modified at the 5′ end of the strand a*b*, the quenching BHQ is modified at the 3′ end of the strand b, and the input DNAzyme Emn binds to the double strand n0/m*n* to replace the strand n0. The strand n0 unlocks the DNAzyme Eab, which in turn reacts with the double strand b/a*b* to release the fluorescent strand b. (B) YES–YES logic circuit corresponding to the logical operation truth table. (C) Native PAGE analysis of the YES–YES logic circuit. The number is marked above each lane, and the top is the DNA strand type for each lane. Lane 1: DNAzyme Emn; lane 2: double stranded n0/m*n*; lane 3: double strand formed by annealing of DNAzyme Eab to strand n0*; lane 4: double stranded b/a*b*; lane 5: double strand formed by annealing of DNAzyme Emn with strand m*n*; lane 6: double stranded n0/n0*; lane 7: strand b, which is the comparison strand of the result; lane 8: double strand formed by annealing of DNAzyme Eab and strand a*b*; lane 9: reaction of YES–YES cascade logic circuit, input 1; and lane 10: YES–YES cascade input logic, no input reaction, Enter 0. The concentration ratio between the strands is 1 : 1. (D) The normalized fluorescence intensity curve of the YES–YES logic circuit, the solution concentration is 0.4 μm, and the sampling scan interval is set to 13 s for a total of 300 cycles. (E) YES–YES logic curve normalized fluorescence curve of fluorescence intensity as a function of Mg^2+^ concentration.

To test the performance of the cascaded circuit, we first established a two-stage YES cascade module. The output of the previous YES logic circuit is the key to turn on the lower YES circuit. Fig. 3A shows the reaction principle of the cascading YES circuit. The E6-type DNAzyme Emn is bound to a double-stranded substrate with a one-sided toehold, releasing a single stranded n0. The input E6-type DNAzyme Eab recognition arms of the downstream YES logic circuit is combined with a single DNA strand and is in a locked inactive state. The single strand released by the upstream reaction can displace the input E6-type DNAzyme of the downstream reaction. Then, the reaction of the second step is performed, and finally, the signal is generated. Fig. 3B shows the truth table of the YES–YES logic circuit. The main problem that was resolved by this module is leakage. The E6-type DNAzyme leaked at the first level of input. Due to the specificity of the E6-type DNAzyme structure, the recognition arm n is identical to the n0 base sequence, which causes the input to directly unlock the inactive E6-type DNAzyme. To resolve this problem, we extended the 5′ end of n0 by three bases. The second leak lies in the way in which the E6-type DNAzyme is locked in an inactive state. Originally designed n0* was combined from the recognition arm along the conservative domain, and the leakage reached 50%. This leak is resolved by changing the lock mode. And the specific method is to combine n0 with the recognition arms.

The results of PAGE observation by gel electrophoresis (Fig. 3C) showed that the lane 7 and lane 8 lanes were the contrast bands of the DNA strands generated in the last step, and the lanes 9 and 10 were input reaction band and no input reaction band, respectively. Because the eighth lane product is similar to the inactivated DNAzyme, these were not compared. Only the input reaction produces the β-strand (lane 7), thus verifying that the YES–YES structure cascade has been successful, and a reference is provided for the multi-level cascade of the YES logic gate. The fluorescence curve (Fig. 3D) also shows that the reaction results are expected, the input reaction curve rises steadily (Fig. 3D, curve 2), and the fluorescence intensity of the reaction without input has substantially no obvious fluctuations (Fig. 3D, curve 1). Fig. 3E shows the contrast in the Mg^2+^ concentration gradient. Although the ion concentration decreased to 1/8, the final fluorescence intensity was still greater than 50% of the original, and the distribution range was not so wide. As with a single YES logic gate, the perception of the concentration changes in Mg^2+^ is not as sensitive as the AND gate. The effect of input strand concentration on results is shown in the ESI (Fig. S6†).

### 2.4. Two-stage YES–AND cascade circuit

**Fig. 4:**
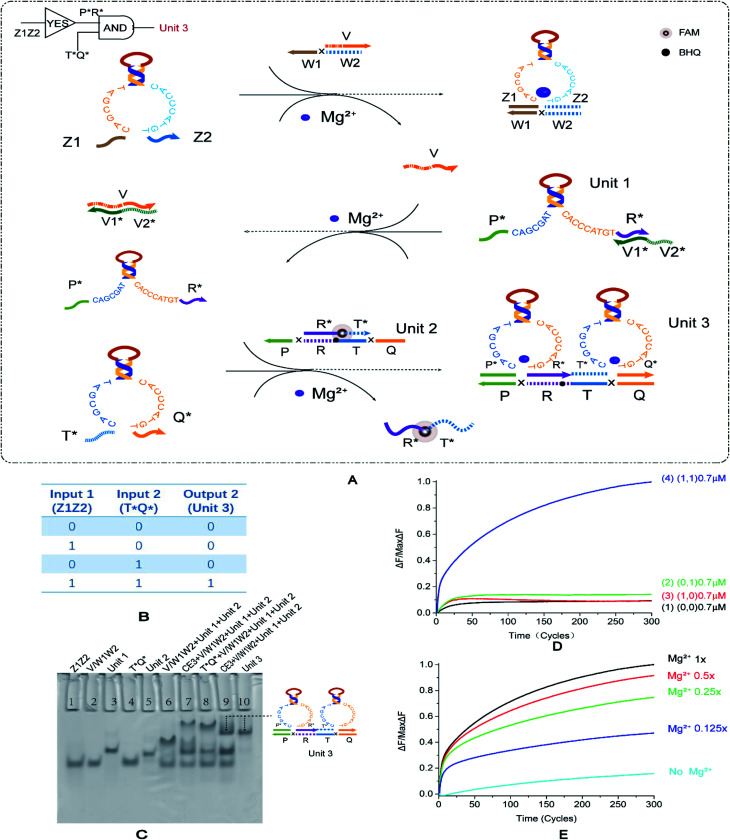
(A) Reaction schematic diagram of the YES–AND cascade logic circuit. The fluorophore FAM is modified in the middle of the strand R*T*, the quenching BHQ modification is in the middle of the strand PRTQ, and the input DNAzyme Z1Z2 binds to the double-stranded V/W1W2 to replace the strand V. Strand V unlocks DNAzyme P*R*. At this point, DNAzymes P*R* and T*Q* are then reacted with the double-stranded R*T*/PRTQ to release the fluorescent strand R*T*. (B) YES–AND logic circuit corresponding to the logical operation truth table. (C) Native PAGE analysis of the YES–AND logic circuit. The number is marked above each lane, and the top is the DNA strand type for each lane. Lane 1: strand Z1Z2; lane 2: double-stranded V/W1W2; lane 3: double strand formed by annealing of DNAzyme P*R* with strand V1*V2*; lane 4: DNAzyme T*Q*; lane 5: double-stranded R *T*/PRTQ; lane 6: YES–AND logic with no input, is (0, 0); lane 7: reaction without input DNAzyme Z1Z2, is (1, 0); lane 8: the reaction without input DNAzyme T*Q* is (1, 0); lane 9: the reaction of YES–AND cascade logic is (1, 1); and lane 10: structure formed by annealing of DNAzyme P*R*, T*Q* and strand PRTQ. The concentration ratio between the strands is 1 : 1. (D) The normalized fluorescence intensity curve of the YES–AND logic circuit, the solution concentration is 0.7 μm, and the sampling scan interval is set to 2 min for a total of 300 cycles. (E) Normalized fluorescence curve of fluorescence intensity as a function of Mg^2+^ concentration in the YES–AND logic circuit.

To explore the circuit diversity of the cascade, we built a YES–AND cascade logic. According to the experience of the YES–YES logic gate, we chose to adjust the double-stranded substrate V/W1W2, extend the 5′ end of the short strand by three bases. We use the DNA strand V1*V2* to lock the E6-type DNAzyme P*R* input in the downstream reaction to form the composite structure Unit 1. This structure is temporarily unable to bind to specific substrates Unit 2. In the first layer of logic gate reactions, E6-type DNAzyme Z1Z2 binds to the double strand to produce a single DNA strand v. This DNA single strand v releases the locked E6-type DNAzyme P*R*. At this point, the recognition arm of E6-type DNAzyme P*R* is exposed and can be used as the input of the second-level AND logic gate together with E6-type DNAzyme T*Q*. The two E6-type DNAzyme input strands of the second level logic gate are combined with their specific double-stranded substrates Unit 2 from two directions to generate the result of the second step, and signal transmission is completed. The truth table of the YES–AND cascade circuit is shown in Fig. 4B.

The results of gel electrophoresis are shown in Fig. 4C, and the lane 6, lane 7, lane 8, and lane 9 are (0, 0), (1, 0), (0, 1), and (1, 1), respectively. The lane 10 is a comparison of results. Only (1, 1) produced the corresponding output. Therefore, the results of PAGE prove that the YES–AND cascade we designed is successful. The fluorescence curve (Fig. 4D, curve 4) also verifies the feasibility of the YES–AND logic cascade. The Mg^2+^ concentration gradient curve (Fig. 4E) slightly differs from other logic circuits. The other logic circuits have a uniform distribution interval at an ion concentration of 12.5 mM and 1/2, 1/4, and 1/8 of 12.5 mM. YES–AND decreases the distribution interval at a higher concentration, indicating that it is sensitive to low concentrations of Mg^2+^. However, when the concentration was 1/2 of 12.5 mM, the reaction was nearly saturated, and thus the fluorescence gap was small. The effect of input strand concentration on results is shown in the ESI (Fig. S7†).

### 2.5. Self-catalytic DNA circuit

**Fig. 5:**
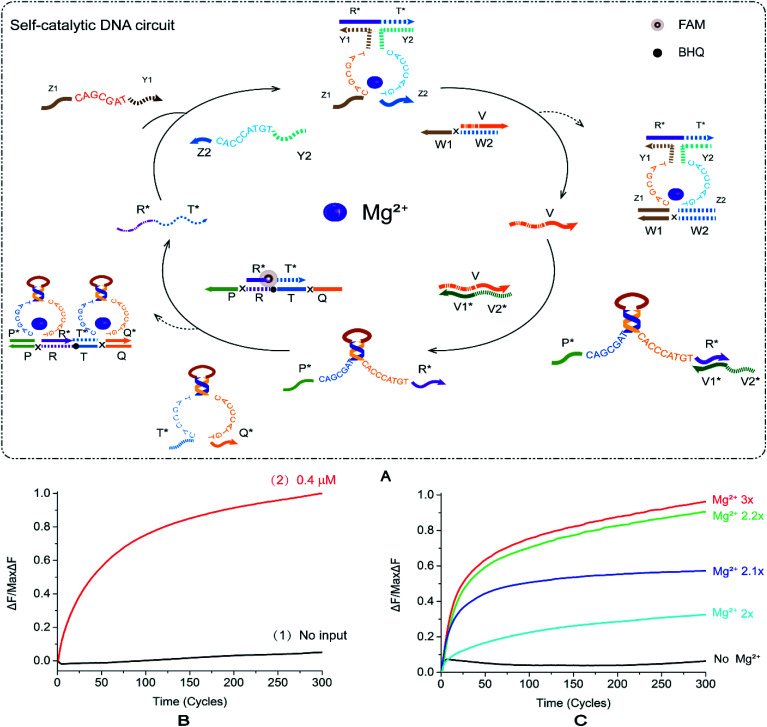
(A) Schematic diagram of the reaction of the self-catalytic DNA circuit. The fluorophore FAM is modified in the middle of the R*T* in the double-stranded R*T*/PRTQ, the quenching BHQ modification is in the middle of the strand PRTQ, and the input R*T* binds to the strands Z1Y1 and Z2Y2 to form a DNAzyme structure, with the double-stranded V/W1W2 combines to displace the strand V. Strand V unlocks DNAzyme EP*R*. At this point, DNAzymes EP*R* and ET*Q* are then reacted with the double-stranded R*T*/PRTQ to release the fluorescent strand R*T*. (B) Normalized fluorescence intensity curve of the self-catalytic DNA circuit; the solution concentration was 0.4 μm, and the sampling scan interval was set to 50 s for a total of 300 cycles. [R*T*] : [Z1Y1] : [Z2Y2] : [V/W1W2] : [V1*V2*/P*R*] : [T*Q*] : [R*T*/PRTQ] = 1.2 : 1 : 1 : 1 : 1 : 1 : 1. (C) Normalized fluorescence curve of fluorescence intensity of DNA autocatalytic circuit as a function of Mg^2+^ concentration.

In order to explore more implementation methods of logic circuits, investigate the feasibility of autocatalysis under this strategy and improve the efficiency of catalytic circuits, we constructed a self-catalytic DNA circuit based on feedback mechanism by modifying the YES–AND cascade logic circuit. In Fig. 5, the input strand of the self-catalytic DNA circuit is a single DNA strand, R*T*, which can be combined with the other two single strands Z1Y1 and Z2Y2 to form a E6-type DNAzyme. R*T* reacts with the double-stranded substrate V/W1W2 under the control of Mg^2+^ to release the DNA single-strand v. The DNA single strand v reacts with the stranded E6-type DNAzyme P*R* to render it active. At this time, E6-type DNAzyme P*R* and E6-type DNAzyme T*Q* react with a specific double-stranded substrate R*T*/PRTQ, and the double-stranded substrate R*T*/PRTQ has a toehold at both sides. E6-type DNAzymes bind toehold from both sides to replace the DNA single strand R*T*. Thus, the strand R*T* is released and can continue to form a new E6-type DNAzyme with the other two double strands, participating in the next round of reaction, and then forming a self-catalytic DNA circuit system.

Fig. 5B shows that in the absence of R*T*, the fluorescence intensity increases only in a small range (Fig. 5B, curve 2), and the leakage is <0.1. After the addition of R*T*, the fluorescence energy rises quickly and stabilizes (Fig. 5B, curve 1). The fluorescence curve intuitively shows that the new mode of action of E6-type DNAzyme is valid in catalytic logic circuits. We added 10× TAE/Mg^2+^ buffer with a concentration of 125 mM to the tube of reaction system according to 1/10 of the total volume of the experimental reaction. All other things being equal, we recorded the change of Mg^2+^ concentration between 0 and 10× on the fluorescence intensity (Fig. S8B†). The fluorescence reaction experiment revealed that when the concentration exceeds 2.2×, the fluorescence of the DNAzyme self-catalytic circuit reaches saturation, and the signal intensity fluctuates within a small range without significant changes. When the Mg^2+^ concentration is lower than 2.2×, the fluorescence intensity exhibited a gradient change (Fig. 5C). As the ion concentration decreases, the fluorescence intensity gradually decreases. In the Mg^2+^-free reaction, the curve smoothly fluctuates and there is no significant increase. It proves that the self-catalytic DNA circuit is most sensitive to the low concentration range of Mg^2+^ compared to the aforementioned circuits.

Our experiments have demonstrated the reliability of E6-type DNAzyme conformational changes and the novel regulation of circuit action through the recognition arms. The entire reaction took a total of four hours, which simplifies the reaction process and improves overall efficiency compared to the self-catalytic DNA circuits constructed by Jing Yang and Ranfeng Wu using E6-type DNAzymes as tools.^29^ This exploration of regulating DNAzyme interaction with double strands has brought us new thinking. In the future, our work is to continue to study the properties of DNAzyme and try to use DNAzyme to participate in the cleavage of double-stranded substrates. If this strategy is feasible, the results in the field of DNAzyme research will be more abundant.

## 3 Experimental

### 3.1. Materials of DNA strands

All DNA strands used herein were purchased from Sangon Biotech (Shanghai, China). The unmodified DNA strand was purified by PAGE, and the DNA strand with the fluorophore and the quencher group modification was purified by high-performance liquid chromatography (HPLC). The specific base sequence is listed in the ESI (Table S1†). All DNA strands were added to the corresponding pure water to prepare a 100 μM stock solution according to the experimental requirements. The concentration was measured using a NanoDrop 2000 spectrophotometer (Thermo Fisher Scientific Inc. USA), and the absorbance was recorded at a wavelength of 260 nm.

### 3.2. Preparation of DNA circuits

The strands in the logic circuits were formed by mixing several DNA strands during slow annealing. We added the DNA strand to 1× TAE/Mg^2+^ buffer {40 mM Tris acetate, 20 mM glacial acetic acid, 2 mM EDTA2Na·2H_2_O, 12.5 mM [Mg(AC)_2_]·4H_2_O, pH 8.0} in the proportions required for the experiment. The DNA strand was added to 1× TAE/Mg^2+^-free buffer (40 mM Tris acetate, 20 mM glacial acetic acid, 2 mM EDTA2Na·2H_2_O, pH 8.0) in the Mg^2+^-free experiment. The DNA strand was added to a final concentration of 8 μM, and the total volume of the solution was 50 μL. The mixture was allowed to react at 95 °C for 5 min, followed by a temperature drop of 0.5 °C min^−1^, and after 146 cycles, the temperature was lowered to 22 °C to complete the annealing. Subsequently, the logical structure was added to an EP tube and placed in a thermocycler at 25 °C for reaction.

### 3.3. Gel electrophoresis experiment

In the electrophoresis experiment, the concentration of the solution in the lane was 1 μM, and the reaction system was 30 μL. That is, the system sample amount is 30p. The environment of the solution was 1× TAE/Mg^2+^ buffer or 1× TAE/Mg^2+^-free buffer depending on the experimental requirements. The electrophoresis instrument used in the experiment was the DYY-6D electrophoresis instrument of Beijing Liuyi Company. We added the experimental logic circuit and the desired comparison strand to the EP tube and reacted at 25 °C for more than one hour. The solution at the end of the reaction was mixed with 60% of glycerol 6 μL. The constant voltage was 70 V and the electrophoresis time was 2 hours and 40 minutes.

### 3.4. Fluorescence experiment

In the fluorescence intensity detection of logic gates (including YES, OR, and AND), cascade circuits and catalytic circuits, the DNA strand concentration ranged from 0.3 μM to 0.7 μM due to specific experimental differences, and the solution volume was 30 μL. The buffer used for the solution was 1× TAE/Mg^2+^ buffer or 1× TAE/Mg^2+^-free buffer. The fluorescence intensity of the solution without the input strand was collected as the initial point in the experiment. The input strand was then added, and changes in fluorescence intensity were continuously recorded, with sampling intervals ranging from 6 s to 2 min. The instrument used in the fluorescence experiment was an Agilent Mx3005P real-time polymerase strand reaction (PCR) system.

## 4 Conclusions

In summary, we constructed a logical operating system that regulates DNA circuits by DNAzyme conformational changes. The focus of this system is to change the single mode of DNAzyme which can only transmit signals by cutting single strand substrates. DNAzyme has a conformational change under the control of Mg^2+^. The recognition arms at both sides of the DNAzyme can complete strand displacement with the double-stranded substrate and release the product. This regulatory strategy extends the way DNAzyme transmits signals. We built basic logic gates, including YES gate, AND gate, and OR gate. In addition, a double-layer cascade logic circuit and a self-catalytic DNA circuit were also constructed. The successful establishment of these circuits provides evidence for the success of regulatory strategy. And this is a general strategy for DNA computing. On the one hand, we changed the traditional way of DNAzyme to transmit signals, simplified the complexity of experimental operations, shortened the time of logical operations, and improved the efficiency of signal transmission at the molecular level. At the same time, the RNA need not be modified on a specific substrate. This simplifies the reaction conditions while reducing material costs. The success of these basic logic operation devices laid the foundation for the construction of larger-scale circuits and provided a new way to promote the development of DNA computing. We will continue to explore this regulatory strategy in combination with other technologies (such as G-quadruplex, Origami, gold nanoparticles, *etc.*) in the future. This will enrich the regulatory methods of biological computing and promote the development of DNA nanotechnology.

## Conflicts of interest

There are no conflicts to declare.

## Supplementary Material

RA-010-D0RA00115E-s001
