# Supplementary material for: DNA circuits driven by conformational changes in DNAzyme recognition arms
Source: RSC Adv. 2020 Feb 24;10(13):7956–66. doi: 10.1039/d0ra00115e (PMC9049901; doi:10.1039/d0ra00115e)
Supplement: RA-010-D0RA00115E-s001 [file RA-010-D0RA00115E-s001.pdf]

Electronic Supplementary Material (ESI) for RSC Advances.
This journal is © The Royal Society of Chemistry 2020

Supplementary Information

DNA circuits driven by conformational changes in DNAzyme recognition arms

Xinyi Sun, [a‡] Xuedong Zheng, [b‡] Sue Zhao, [a] Yuan Liu, [c] and Bin Wang, [*a]

[1] Key Laboratory of Advanced Design and Intelligent Computing, Ministry of Education, School of Software Engineering, Dalian University, Dalian 116622, China; E-mail: wangbinpaper@gmail.com

[2] College of Computer Science, Shenyang Aerospace University, Shenyang 110136, China; xuedongzheng@163.com

[3] School of Computer Scicence and Technology, Dalian University of Technology, Dalian 116024, China; E-mail: Liuyuan.dlut@gmail.com

* Correspondence: wangbinpaper@gmail.com; Tel.: +86-0411-87402106 (B.W.); Fax: +86-0411-87403733 (B.W.)

‡ These authors contributed equally to this work.

Contents

Supplementary Information 1
DNA circuits driven by conformational changes in DNAzyme recognition arms 1
Table S1. DNA sequences 3
1 Materials 6
2 Methods 6
2.1 YES Logic gate 6
2.2 OR Logic gate 7
2.3 AND Logic gate 8
2.4 Two-stage YES cascade circuit and YES-AND cascade logic 9
2.4 Self-catalytic DNA circuit 10
2.5 Application of the principle of conformation changes in cleavage reaction 10

Table S1. DNA sequences

Table S1. DNA sequences

Index	Name	Sequence (5' to 3')	Length
1	d0	AGGATGAATGTTT	13
2	E6	GGAGACAGCGATCCGGAACGGCACC CATGTAGGATGAATG	40
3	t*xd*1	CATTCATCCTGGTCTCC	17
4	t*xd*2	CATTCATCCTCGGTCTCC	18
5	t*xd*3	CATTCATCCTTAGGTCTCC	19
6	ORE10	GCGTGCAGCGATAGCGTAGCTCACCC ATGTCCTTAATCTGC	41
7	ORE20	CCTTAATCTGCAGCGATCCGGAACGG CACCCATGTCGTCAAC	42
8	ORt*d*b*	GTTGACGTGGCAGATTAAGGTGGCAC GC	28
9	ORd0	CCTTAATCTGC	11
10	ANDE1	TGACCTCCACAGCGATCCGGAACGGC ACCCATGTATACTCCTCT	44
11	ANDE2	CATCTCATTCAGCGATCCGGAACGGC ACCCATGTACCCACCTAG	44
12	t1*d1*d2*t2	AGGTGGGTGGAATGAGATGAGAGGA GTATGGTGGAGGT	38
13	d1d2	ATACTCCTCTCCATCTCATTC	21
14	Emn	GCGACCTCCCCATACTCCTCTCCATCT TCAGCGATCCGGAACGGCACCCATGT TCTCTC	59
15	n0	ATGGCGACCTCCCCATACTCCTCTCC ATCTTC	32

16	m*n*	GAGAGACGAAGATGGAGAGGAGTAT GGGGAGGTCGC	36
17	n0*	GAAGATGGAGAGGAGTATGGGGAGG TCGCCAT	32
18	Eab	ACCTCCAGCGATCCGGAACGGCACCC ATGTCCATACTCCTCTCCATCTTCTCC	53
19	b	CCATACTCCTCTCCATCTTCTCC	23
20	a*b*	GGAGAAGATGGAGAGGAGTATGGCG GAGGT	30
21	Z1Z2	TAATGACCTCCACAGCGATCCGGAAC GGCACCCATGTTCTCTC	43
22	Z1Y1	TAATGACCTCCACAGCGATCAGCGAT TAACAGAGGAGTAT	40
23	Z2Y2	GAATGAGATGGTTACACCCATGTTCT CTC	29
24	R*T*	ATACTCCTCTCCATCTCATTC	21
25	V	GCCTAATGACCTCCACAGCGAT	22
26	W1W2	GAGAGAATCGCTGTGGAGGTCATTA	25
27	V1*V2*	ATCGCTGTGGAGGTCATTAGGC	22
28	P*R*	TGACCTCCACAGCGATCCGGAACGGC ACCCATGTATACTCCTCT	44
29	T*Q*	CATCTCATTCAGCGATCCGGAACGGC ACCCATGTACCCACCTAG	44
30	PRTQ	AGGTGGGTGGAATGAGATGAGAGGA GTATGGTGGAGGT	38
31	AND1	TCCTCTTATTCTTCT	15
32	AND2	CTCCTCATATTCTTCT	16
33	AND3	TCCTCTCTACTCTTCT	16
34	AND4	TCCTCTCTAACTCTTCT	17

35	AND5	TCCTCTCTAAACTCTTCT	18
36	AND6	TCCTCTCAGAACTCTTCT	18
37	AND7	TCCTCTCAGCTCTTCT	16
39	CF	CTCTTCAGCGATCCGGAACGGCACCC<br>ATGTATGATGCTTC	40
39	C*F*	GAAGCATCATT/rA/GGAAGAG	19
40	F	ATGATGCTTGTTT	13

All of the sequences used in this work were designed using Nupack.

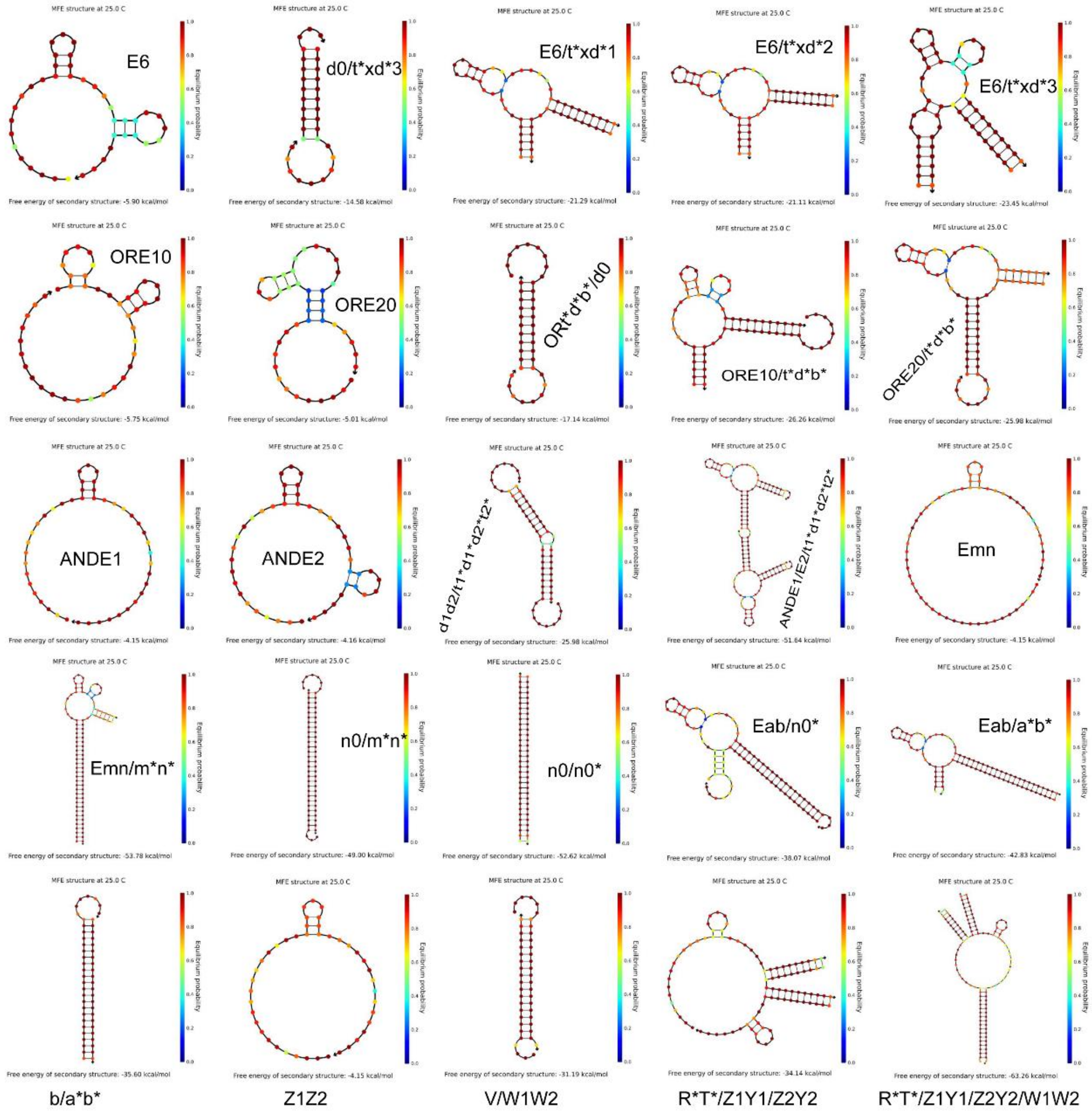

Figure S1. Nupack simulation for sequences in Table S1.

1 Materials

All DNA strands (Table S1) used herein were purchased from Sangon Biotech (Shanghai, China). The unmodified DNA strand was purified by PAGE, and the DNA strand with the fluorophore and the quencher group modification was purified by high-performance liquid chromatography (HPLC). In the gel electrophoresis experiment, we used 12% PAGE (5.89mL pure water, 3 mL of mother liquor of acrylamide at a concentration of 40% (300mL pure water, 190 g of acrylamide and 10 g of N, N' -methylenebis), 1 mL 10x TAE, 100 uL ammonium persulfate (APS), 10 uL N, N, N', N'-Tetramethylethylene diamine (TEMED)). The gel duration is 40 min. The results of the staining were performed using Stains All (0.1 g of Stains All powder, 450 mL of formamide, 550 mL of pure water). The pure water(18.2MΩ/cm) used in all the experimental protocols was obtained from the Milli-Q purification system. Other chemicals were of reagent grade and were used without further purification.

2 Methods

2.1 YES Logic gate

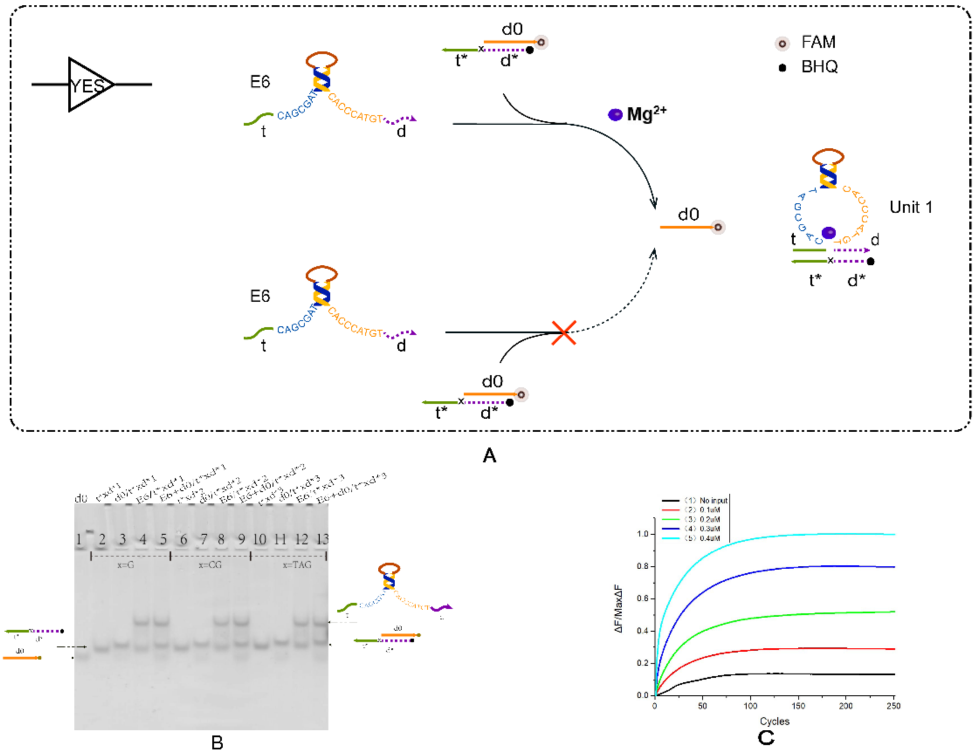

Figure S2. (A) YES logic gate comes in two forms, one with a switch and one without a switch. That is, no Mg^{2+} are added to the solution, and Mg^{2+} are added to one solution. (B) PAGE in the absence of Mg^{2+} in the solution. (C) YES logic gate input strand concentration gradient comparison, input strand concentration is 0.1uM, 0.2uM, 0.3uM, 0.4uM, and the solution concentration at no input is 0.4uM. Sampling scan interval time was set to 6 s, for a total of 251 cycles.

In the basic logic gate, we performed two gel electrophoresis experiments in order to verify the effect of

Mg^{2+} on the experiment. One has the presence of Mg^{2+}, one without adding Mg^{2+}. As shown in Figure S1B, we can see from the 4th, 8th, and 12th lanes that if there is no Mg^{2+}, the E6 strand and t*d* can not form a double-stranded state. It can be seen from the 5th, 9th, and 13th lanes that in the absence of Mg^{2+}, the input strand E6 does not react with the double-stranded substrate d0/t*d*, and both are in a free state in solution. This verifies the total switching effect of the Mg^{2+} on the logic gates. Without the presence of Mg^{2+}, the reaction could not proceed even with the input strand. As shown in Figure S1C, this is the different fluorescence response exhibited by the YES logic gate for different input strand concentrations.

2.2 OR Logic gate

After the YES logic gate succeeds, we have designed the OR logic gate. As shown in Figure S2A, DNAzymes bind to specific double-stranded substrates from two directions, and half of them have the same recognition domain. This ensures that both inputs can replace the d0 strand. Figure S2B is the PAGE of the OR logic gate with Mg^{2+}. The third and fourth lanes are the result comparison. The 4th, 5th, and 6th lanes are (1,0), (0,1), and (1,1), respectively. The result of PAGE reflects that the OR logic gate is established. Figure S2C is a PAGE without Mg^{2+}. It can be seen from the sixth lane in the figure that in the absence of Mg^{2+}, the two inputs are not combined with the substrate, indicating that Mg^{2+} does function as a switch.

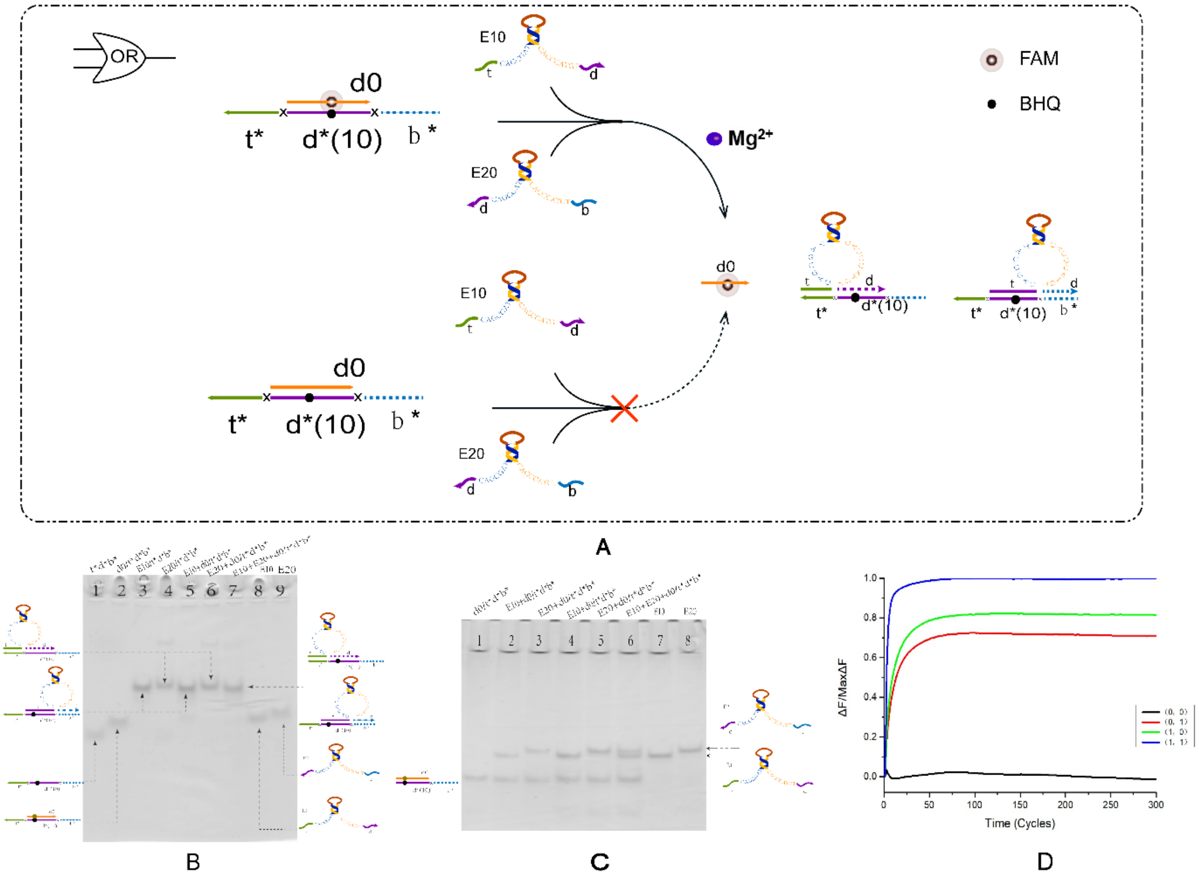

Figure S3. (A) Schematic diagram of the OR logic gate principle. (B) PAGE with Mg^{2+}: lane 1: t*d*b*; lane 2: double stranded d0/t*d*b*; lane 3: double stranded E10/t*d*b*; lane 4: double-stranded E20/t*d*b*; lane 5: E10+double-stranded d0/t*d*b*; lane 6: E20+double-stranded d0/t*d*b*; :E10+E20+ double strand d0/t*d*b*; lane 8: DNAzyme E10; lane 9: DNAzyme E20. (C) PAGE without

Mg^{2+} ion. Lane 1: double strand d0/t*d*b*; lane 4: E10 + double strand d0/t*d*b*; lane 5: E20 + double strand d0/t *d*b*; lane 6: E10 + E20 + double stranded d0/t*d*b*; lane 7: DNAzyme E10; lane 8: DNAzyme E20. (D) OR logic gate fluorescence intensity curve. The duration is 30 minutes and the scanning interval is 6 s.

2.3 AND Logic gate

Initially, the AND logic solved the problem of low binding rates between two input strand DNAzymes and double-stranded substrates. As shown in Figure S3, the sixth, seventh, and eighth tracks are (1,0), (0,1), (1,1). It can be seen that there are a large number of input strands remaining, which cannot be ideal result. This problem is solved by increasing the content of CG bases in the DNAzyme recognition domain. Then, new problems arise. Although the input strand is combined with a double-stranded substrate, it produces less output. We began to explore the method of combining double-stranded substrates. We tried to mismatch the base and base bubbling in order to make the output strand easier to drop. It was confirmed by PAGE that when there is a bubbling of a base length, the number of double-stranded substrates formed is the largest, and there is no remaining. At this point, the output strand is easy to fall, and the leakage is minimal at this time. In other cases, some of the strands do not form a double strand, which is likely to cause leakage.

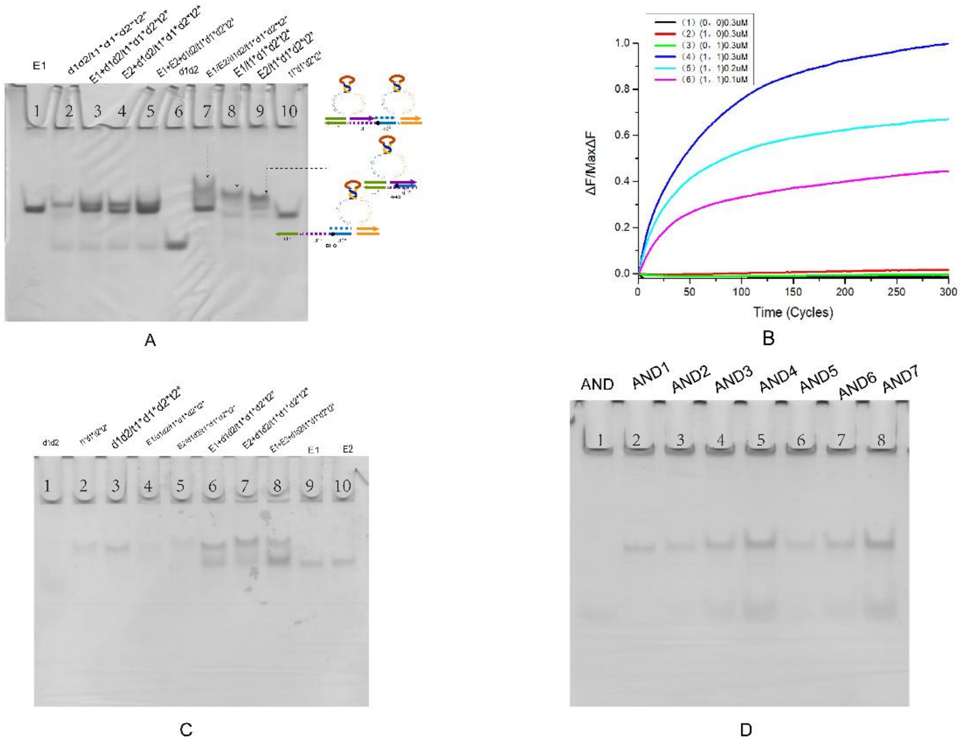

Figure S4. (A) PAGE without Mg^{2+}. The 2nd, 3rd, 4th, and 5th tracks are (0,0), (1,0), (0,1) (1,1). (B) AND logic gate fluorescence intensity curve. The input strand concentrations were 0.1 uM, 0.2 uM, 0.3 uM. The other DNA strand concentrations were 0.3 uM. And the sampling scan interval is set to 10 s for a total of 300 cycles. (C) Failure AND logic gate PAGE. (D) Select the PAGE of the double-stranded substrate binding mode. Lane 1: Comparison of output single strands; Lane 2: one base bubbling; Lane 3: 2 bases bubbling; Lane 4: 3 bases bubbling; Lane 5: 4 bases Bubbling; lane 6: one base mismatch and bubbling; lane 7: 3 base mismatch; lane 8: 4 base mismatch.

2.4 Two-stage YES cascade circuit and YES-AND cascade logic

In the process of inactivating the DNAzyme of the second step, the YES logic circuit starts to select the blocking domain on one side and the conserved domain in part. However, as shown in Figure S4AB, the leakage of the fluorescent image is very serious. Increasing the number of bases bound to DNAzymes does not solve this problem. Finally, we changed our mindset to hide most of the recognition domain of DNAzymes in the site of double-stranded binding. This recognition domain is unable to react with the double-stranded substrate, thus reducing the occurrence of leakage. This makes the cascade circuit successful.

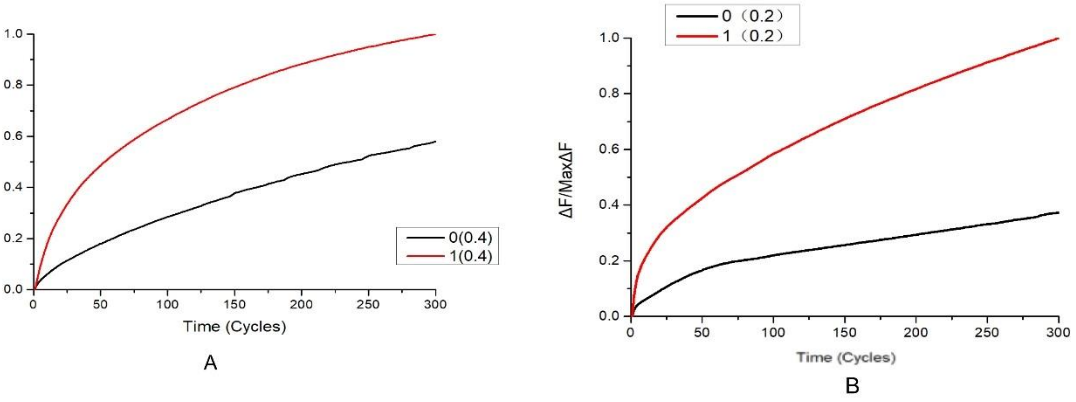

Figure S5. (A) A fluorescent map in which the recognition domain of one side and a part of the conserved domain are blocked. (B) In the case where the recognition domain of one side and a part of the conserved domain are blocked, the fluorescence map after the number of binding bases is increased.

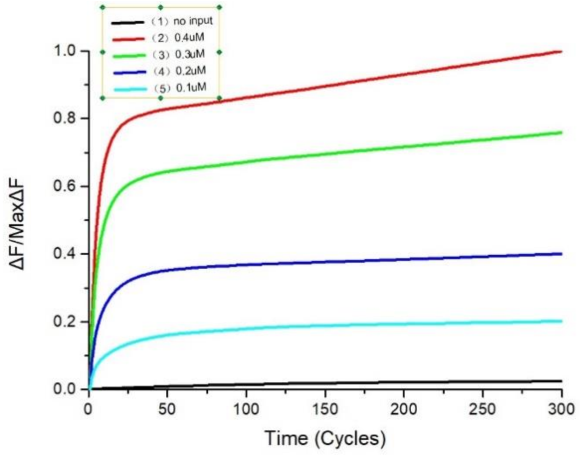

Figure S6. Input strand concentration gradient comparison. The input strand concentrations were 0.1 uM, 0.2 uM, 0.3 uM, 0.4 uM. The other DNA strand concentrations were 0.4 uM. And the sampling scan interval is set to 13 s for a total of 300 cycles.

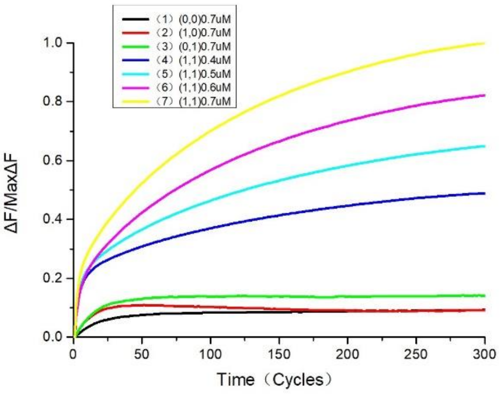

Figure S7. Input strand concentration gradient comparison of the YES-AND logic circuit. The input strand concentrations were 0.4 uM, 0.5 uM, 0.6 uM, 0.7 uM. The other DNA strand concentrations were 0.7 uM. And the sampling scan interval is set to 2 min for a total of 300 cycles.

2.4 Self-catalytic DNA circuit

Adjust the Mg^{2+} concentration in the buffer to the standard data 12.5 mM, which is defined as 10x. For the DNA autocatalytic circuit, in the process of making the Mg^{2+} concentration gradient, it is found that the fluorescence intensity of the circuit system falls within a range when the fluorescence intensity is higher than 2.2x. That is, the concentration of Mg^{2+} reaches a saturated state. It is indicated that the presence of Mg^{2+} in the DNA autocatalytic circuit is more sensitive than other circuits.

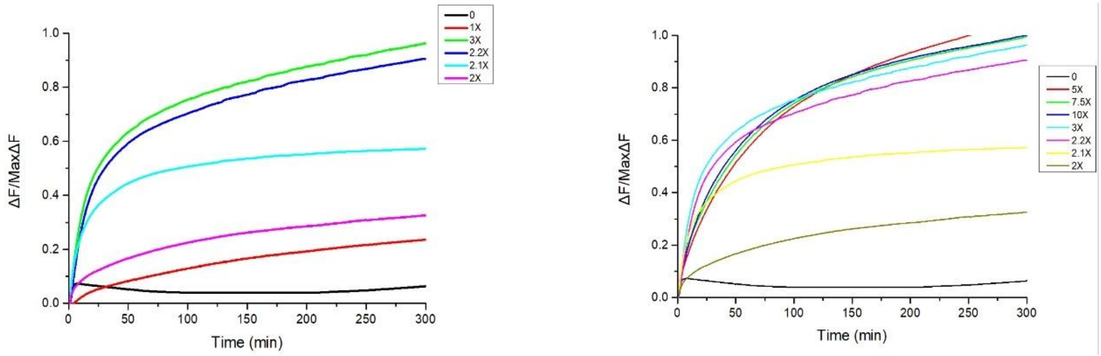

A B

Figure S8. (A) Mg^{2+} concentration 0-3x fluorescence intensity curve. (B) Mg^{2+} concentration 0-10x fluorescence intensity curve.

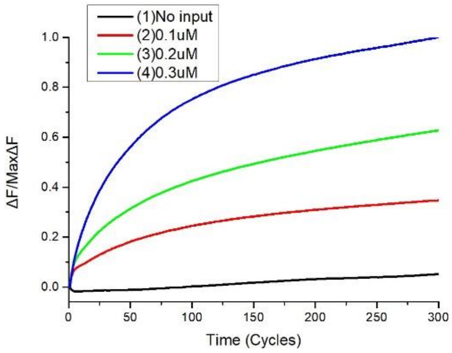

Figure S9. Input strand concentration gradient comparison of the Self-catalytic DNA circuit. The input strand concentrations were 0.1 uM, 0.1 uM, 0.1 uM. The other DNA strand concentrations were 0.3 uM. And the sampling scan interval is set to 50s for a total of 300 cycles.

2.5 Application of the principle of conformation changes in cleavage reaction

In order to verify whether the principles proposed in this study are applicable to DNAzyme cutting substrates located in double strands, we designed a YES logic gate based on DNAzyme cleavage. The experimental results prove that DNAzyme can cut the substrate that is not fully exposed in the double strand.

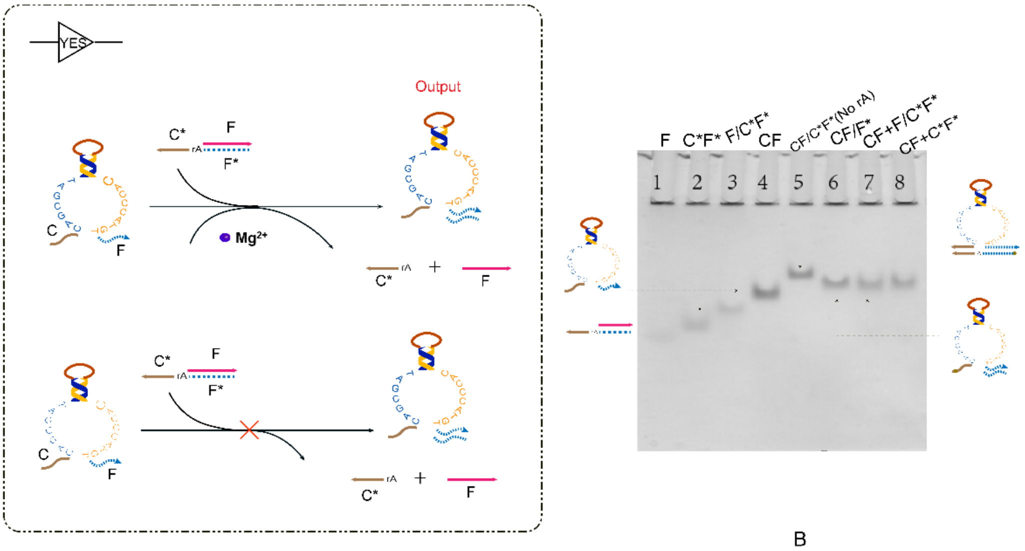

A

B

Figure S10. (A) YES logic gate for cutting the substrate in double strands using the principle of conformational change. (B) Native PAGE analysis of the YES logic gate.
